# Loss of heterozygosity on chromosome 22 in ovarian carcinoma is distal to and is not accompanied by mutations in NF2 at 22q12.

**DOI:** 10.1038/bjc.1994.418

**Published:** 1994-11

**Authors:** P. Englefield, W. D. Foulkes, I. G. Campbell

**Affiliations:** University of Southampton, Princess Anne Hospital, Hants, UK.

## Abstract

**Images:**


Br. J. Cancer (1994), 70, 905-907 © Macmillan Press Ltd., 1994

### Loss of heterozygosity on chromosome 22 in ovarian carcinoma is distal to and is not accompanied by mutations in *NF2* at 22q12

P. Englefield[1], W.D. Foulkes[2] & I.G. Campbell[1]

[1]*Obstetrics and Gynaecology, University of Southampton, Princess Anne Hospital, Coxford Road, Southampton, Hants SO15 5YA, UK;* [2]*Human Immunogenetics Laboratory, Imperial Cancer Research Fund, PO Box 123, Lincoln's Inn Fields, London WC2A 3PX, UK.*

**Summary** Frequent loss of heterozygosity (LOH) has been reported on 22q in ovarian carcinoma, implying the presence of a tumour-suppressor gene. The neurofibromatosis type 2 gene (*NF2*) at 22q12 is a plausible candidate. Analysis of 9 of the 17 exons of *NF2* by single-strand conformational polymorphism (SSCP) in 67 ovarian carcinomas did not detect any somatic mutations, suggesting that *NF2* is not involved in the pathogenesis of ovarian carcinoma. LOH data support this conclusion and that the putative tumour-suppressor gene lies distal to *NF2*, beyond D22S283.

Cytogenetic and allele loss studies point to the involvement of a chromosome 22 tumour-suppressor gene in a variety of malignancies. Loss of 22q markers has been observed in 11-38% of breast cancers (Sato *et al.*, 1990; Chen *et al.*, 1992; Lindblom *et al.*, 1993), 21-50% of colon carcinomas (Miyaki *et al.*, 1990; Okamoto *et al.*, 1988) and 33% of hepatocellular carcinomas (Takahashi *et al.*, 1993). In ovarian carcinoma, loss of 22q markers has been reported at frequencies of up to 71% (Cliby *et al.*, 1993; Dodson *et al.*, 1993), although others have reported lower frequencies in the range of 25% (Sato *et al.*, 1991; Yang-Feng *et al.*, 1993; Osborne & Leech, 1994). These studies have not defined a minimum region of LOH. However, with the exception of hepatocellular carcinomas, the 22q losses are consistent with deletion of the region containing *NF2* on 22q12 (Rouleau *et al.*, 1993; Trofatter *et al.*, 1993) and this therefore may be the target for the LOH.

Neurofibromatosis type 2 (NF2) is an autosomal dominant syndrome which predisposes to schwannomas, meningiomas, ependymomas and juvenile cataracts. LOH for DNA markers on chromosome 22 has been found in both hereditary and sporadic tumours associated with the NF2 phenotype (Sanson *et al.*, 1993), suggesting that *NF2* is acting as a tumour-suppressor gene. *NF2* is expressed in a variety of non-central nervous system tissues and therefore could be implicated in tumours arising from these tissues. Although ovarian carcinoma is not part of the NF2 syndrome, somatic mutations in the tumour-suppressor gene *NF1*, for example, have been reported in tumour types not usually associated with the hereditary syndrome (Li *et al.*, 1992). The strong genetic evidence for the presence of a tumour-suppressor gene on 22q prompted us to undertake a mutation analysis of *NF2* in ovarian carcinomas and, if appropriate, a deletion analysis to determine the smallest region of loss.

### Materials and methods

### *Tumour specimens and DNA extraction*

Tumour and blood samples were obtained from 67 patients undergoing primary surgery for ovarian carcinoma. Thirty-eight of the samples have been described previously and have been the subject of extensive molecular studies (Foulkes *et al.*, 1993*a-c*, 1994; Allan *et al.*, 1994). The remaining 29 samples were collected from hospitals in and around Southampton. The 67 tumours studied included 46 serous tumours, 11 mucinous, two mixed Müllerian, seven endometrioid and one granulosa cell tumour. DNA was extracted from the tumours and blood as described by Foulkes *et al.* (1993*a*).

### *Polymerase chain reactions (PCRs)*

PCRs were performed on 10-200 ng of genomic DNA in a reaction volume of 20 µl with the inclusion of 1 µCi of [α-$^{32}$P]dCTP (Foulkes *et al.*, 1993*c*) using the published PCR cycle conditions.

### *SSCP analysis*

*NF2* exons were amplified with primers within surrounding intronic sequence. Details of these primers, including the regions amplified by each set and the PCR conditions used, have been reported by Twist *et al.* (1994). Samples were prepared for single-strand conformational polymorphism analysis as described previously (Campbell *et al.*, 1994) and electrophoresis carried out at room temperature in 6% non-denaturing acrylamide gels in the presence of 5% and 10% glycerol.

### *Microsatellite analysis*

The chromosome 22q primers used were D22S430 (Sainz *et al.*, 1993), D22S282, D22S283 and D22S274 (Weissenbach *et al.*, 1992). PCR products were resolved on 8% non-denaturing polyacrylamide gels.

### Results

Nine of the 17 coding exons of *NF2* (exons 2, 5, 7-12 and 15) were examined for the presence of somatic mutations. The SSCP analysis was biased toward more 5′ exons since the N-terminal region of the merlin protein is more frequently associated with mutations (Deprez *et al.*, 1994), however no mutations were detected in any of the 67 ovarian carcinomas. The sensitivity of SSCP analysis has been shown to be related to the size of the PCR product (Sheffield *et al.*, 1993). In our study, this was 188 bp, and we therefore estimate the SSCP analysis sensitivity to be 70-75%.

In view of the absence of detectable *NF2* mutations we undertook a preliminary allelotype analysis of 37 ovarian carcinomas (from the collection described by Foulkes *et al.*, 1993*a-c*) in order to identify tumours with partial loss of 22q, since previous studies have not enabled an assignment of the gene to any particular region on chromosome 22. All four 22q markers used map distal to *NF2*, with the most proximal marker, D22S430, located in a region approximately 300 kb distal to *NF2*. The relative genetic and

Correspondence: I.G. Campbell.
Received 14 June 1994; and in revised form 12 July 1994.

**Figure 1** Allelic deletion pattern for ovarian tumours 28, 72 and 91 showing partial loss of chromosome 22q. Unshaded boxes, no LOH; black filled boxes, LOH; grey filled boxes, constitutional homozygosity. For each informative locus, the autoradiograph of the normal (N) and tumour (T) DNA PCR is shown to the right. A genetic linkage map of the four markers used is shown to the right of a karyogram of chromosome 22 and is based on published maps (Matise *et al.*, 1994; Buetow *et al.*, 1994). The inter-locus distances are in centimorgans. The position of the *NF2* gene is indicated just proximal to D22S430.

cytogenetic positions of these markers are shown in Figure 1. Twenty-three of 32 informative tumours (72%) showed loss of one or more 22q markers. In three of these tumours partial loss of 22q was observed (Figure 1). In tumour 28, complete LOH was observed with D22S274 and D22S282, while only partial LOH was seen with D22S283. The finding was consistently found on repeated testing and therefore might be accounted for by clonal variation. In tumour 72, heterozygosity is clearly maintained with D22S283 and completely lost with the more distal markers. In tumour 91, there was maintenance of heterozygosity with the most proximal marker D22S430 but clear loss with the distal markers D22S283 and D22S274.

### Discussion

LOH data suggest that a tumour-suppressor gene relevant in ovarian, breast and colon carcinomas resides on chromosome 22, but it is unclear at this stage if the same gene is involved in these and other malignancies. *NF2*, located at 22q12, is a plausible candidate for this gene, but to date somatic mutation analysis has been restricted largely to tumours of the central nervous system (CNS). However, in two reports, mutations were detected in the coding region of *NF2* in a total of 1 of 69 breast carcinomas, 6 of 17 melanomas and 2 of 64 colon carcinomas (Arakawa *et al.*, 1994; Biachi *et al.*, 1994). The significance of the low frequencies of *NF2* mutation in the breast and colon carcinomas is unclear. However, in this study of 67 ovarian carcinomas, the absence of *NF2* mutations supports the conclusion that *NF2* is not involved in the pathogenesis of ovarian carcinoma. Although we have not examined all the *NF2* coding exons, studies in NF2-related tumours (Rouleau *et al.*, 1993; Deprez *et al.*, 1994; Rubio *et al.*, 1994; Ruttledge *et al.*, 1994; Twist *et al.*, 1994) and non-CNS tumours (Arakawa *et al.*, 1994; Biachi *et al.*, 1994) have shown that mutations are distributed throughout the gene with a bias towards more 5′ exons. The distribution of *NF2* mutations is unlikely to differ significantly in ovarian carcinomas, and given our large sample size we consider it improbable that *NF2* is the 22q ovarian carcinoma tumour-suppressor gene.

In our preliminary allelotype study we demonstrated 72% LOH of 22q markers, consistent with the frequencies reported by Cliby *et al.* (1993) and Dodson *et al.* (1993). Three tumours were identified which had retained heterozygosity for markers proximal to *NF2* but showed LOH for more distal markers. Although some somatic deletions might be the result of generalised genomic instability unrelated to tumour pathogenesis, the finding of three tumours with consistent losses distal to *NF2*, coupled with the absence of mutations in the *NF2* gene, support the conclusion that the 22q ovarian carcinoma tumour-suppressor gene lies distal to *NF2*, beyond D22S283. Detailed deletion mapping of 22q in an expanded set of ovarian carcinomas is currently under way to refine the location of this gene.

**Abbreviations:** LOH, loss of heterozygosity; NF2, neurofibromatosis type 2; PCR, polymerase chain reaction; CNS, central nervous system.

This work was funded by a grant from the Medical Research Council. Additional support was provided by the Wessex Medical Trust. We thank Mrs M. Baron and Mrs N. Thomas and the clinicians from hospitals in the Wessex region who provided tumour samples and Dr G. Rouleau for providing the *NF2* primer sequences.

**References**

ALLAN, G.J., COTTRELL, S., TROWSDALE, J. & FOULKES, W.D. (1994). Loss of heterozygosity on chromosome 5 in sporadic ovarian carcinoma is a late event and is not associated with mutations in APC at 5q21-22. *Hum. Mutat.*, **3**, 283-292.

ARAKAWA, H., HAYASHI, N., NAGASE, H., OGAWA, M. & NAKAMURA, Y. (1994). Alternative splicing of the NF2 gene and its mutation analysis of breast and colorectal cancers. *Hum. Mol. Genet.*, **3**, 565-568.

BIACHI, A.B., HARA, T., RAMESH, V., GAO, J., KLEIN-SZANTO, A.J.P., MORIN, F., MENON, A.G., TROFATTER, J.A., GUSELLA, J.F., SEIZINGER, B.R. & KLEY, N.M. (1994). Mutations in transcript isoforms of the neurofibromatosis 2 gene in multiple human tumour types. *Nature Genet.*, **6**, 185-192.

BUETOW, K.H., WEBER, J.L., LUDWIGSEN, S., SCHERPBIER-HEDDEMA, T., DUYK, G.M., SHEFFIELD, C.V., WANG, Z. & MURRAY, J.C. (1994). Integrated human genome-wide maps constructed using the CEPH reference panel. *Nature Genet.*, **6**, 391-393.

CAMPBELL, I.G., NICOLAI, H.M., FOULKES, W.D., STAMP, G.W., ALLAN, G., BOYER, C.M., SENGER, G., JONES, K., BAST, Jr, R.C., SOLOMON, E., TROWSDALE, J. & BLACK, D.M. (1994). A novel gene encoding a B-box protein within the BRCA1 region at 17q21.1. *Hum. Mol. Genet.*, **3**, 589-594.

CHEN, L.C., KURISU, W., LJUNG, B.M., GOLDMAN, E.S., MOORE, II, D. & SMITH, H.S. (1992). Heterogeneity for allelic loss in human breast cancer. *J. Natl Cancer Inst.*, **84**, 506-510.

CILBY, W., RITLAND, S., HARTMANN, L., DODSON, M., HALLING, K.C., KEENEY, G., PODRATZ, K.C. & JENKINS, R.B. (1993). Human epithelial ovarian cancer allelotype. *Cancer Res.*, **53**, 2393-2398.

DEPREZ, R.H.L., BIANCHI, A.B., GROEN, N.A., SEIZINGER, B.R., HAGEMEIJER, A., VAN DRUNEN, E., BOOTSMA, D., KOPER, J.W., AVEZAAT, C.J.J., KLEY, K. & ZWARTHOFF, E.C. (1994). Frequent NF2 gene transcript mutations in sporadic meningiomas and vestibular schwannomas. *Am. J. Hum. Genet.*, **54**, 1022-1029.

DODSON, M.K., HARTMANN, J.C., CLIBY, W.A., DELACEY, K.A., KEENEY, G.L., RITLAND, S.R., SU, J.Q., PODRATZ, K.C. & JENKINS, R.B. (1993). Comparison of loss of heterozygosity patterns in invasive low-grade and high-grade epithelial ovarian carcinomas. *Cancer Res.*, **53**, 4456-4460.

FOULKES, W.D., CAMPBELL, I.G., STAMP, G.W.H. & TROWSDALE, J. (1993*a*). Loss of heterozygosity and amplification of chromosome 11q in ovarian cancer. *Br. J. Cancer*, **67**, 268-273.

FOULKES, W.D., RAGOUSIS, J., STAMP, G.W.H., ALLAN, G.J. & TROWSDALE, J. (1993*b*). Frequent loss of heterozygosity on chromosome 6 in human ovarian carcinoma. *Br. J. Cancer*, **67**, 551-559.

FOULKES, W.D., BLACK, D.M., STAMP, G.W.H., SOLOMON, E. & TROWSDALE, J. (1993*c*). Very frequent loss of heterozygosity throughout chromosome 17 in sporadic ovarian cancer. *Int. J. Cancer*, **54**, 220-225.

FOULKES, W.D., ENGLEFIELD, P. & CAMPBELL, I.G. (1994). Mutation analysis of RASK and the 'FLR exon' of NF1 in sporadic ovarian carcinoma. *Eur. J. Cancer*, **30A**, 528-530.

LI, Y., BOLLAG, G., CLARK, R., STEVENS, J., CONROY, L., FULTS, D., WARD, K., FRIEDMAN, E., SAMOWITZ, W., ROBERTSON, M., BRADLEY, P., McCORMICK, F., WHITE, R. & CAWTHON, R. (1992). Somatic mutations in neurofibromatoses 1 gene in human tumors. *Cell*, **69**, 275-281.

LINDBLOM, A., SKOOG, L., ROTSTEIN, S., WERELIUS, B., LARSSON, C. & NORDENSKJÖLD, M. (1993). Loss of heterozygosity in familial breast carcinomas. *Cancer Res.*, **53**, 4356-4361.

MATISE, T.C., PERLIN, M. & CHAKRAVARTI, A. (1994). Automated construction of genetic linkage maps using an expert system (MultiMap): a human genome linkage map. *Nature Genet.*, **6**, 384-390.

MIYAKI, M., SEKI, M., OKAMOTO, M., YAMANAKA, A., MAEDA, Y., TANAKA, K., KIKUCHI, R., IWAMA, T., IKEUCHI, T., TONOMURA, A., NAKAMURA, Y., WHITE, R., KIKI, Y., UTSUNOMIYA, J. & KOIKE, M. (1990). Genetic changes and histopathological types in colorectal tumors from patients with familial adenomatous polyposis. *Cancer Res.*, **50**, 7166-7173.

OKAMOTO, M., SASAKI, M., SUGIO, K., SATO, C., IWAMA, T., IKEUCHI, T., TONOMURA, A., SASAZUKI, T. & MIYAKI, M. (1988). Loss of constitutional heterozygosity in colon carcinoma from patients with familial polyposis coli. *Nature*, **331**, 273-277.

OSBORNE, R.J. & LEECH, V. (1994). Polymerase chain reaction allelotyping of human ovarian cancer. *Br. J. Cancer*, **69**, 429-438.

ROULEAU, G.A., MEREL, P., LUTCHMAN, M., SANSON, M., ZUCMAN, J., MARINEAU, C., HOANG-XUAN, K., DEMCZUCK, S., DESMAZE, C., PLOUGASTEL, B., PULST, S.M., LENOIR, G., BIJLSMA, E., FASHOLD, R., DUMANSKI, J., DE JONG, P., PARRY, D., ELDRIGE, R., AURIAS, A., DELATTRE, O. & THOMAS, G. (1993). Alteration in a new gene encoding a putative membrane-organizing protein causes neuro-fibromatosis type 2. *Nature*, **363**, 515-521.

RUBIO, M., CORREA, K.M., RAMESH, V., MACCOLLIN, M.M., JACOBY, L.B., VON DEIMLING, A., GUSELLA, J.F. & LOUIS, D.N. (1994). Analysis of the neurofibromatosis 2 gene in human ependymomas and astrocytomas. *Cancer Res.*, **54**, 45-47.

RUTTLEDGE, M.H., SARRAZIN, J., RANGARATNAM, S., PHELAN, C.M., TWIST, E., MEREL, P., DELATTRE, O., THOMAS, G., NORDENSKJÖLD, M., COLLINS, V.P., DUMANSKI, J.P. & ROULEAU, G.A. (1994). Evidence for the complete inactivation of the NF2 gene in the majority of sporadic meningiomas. *Nature Genet.*, **6**, 180-184.

SAINZ, J., NECHIPORUK, A., KIM, U., SIMON, M.I. & PULST, S.M. (1993). CA-repeat polymorphism at the D22S430 locus adjacent to NF2. *Hum. Mol. Genet.*, **2**, 2203.

SANSON, M., MARINEAU, C., DESMAZE, C., LUTCHMAN, M., RUTTLEDGE, M., BARON, C., NAROD, S., DELATTRE, O., LENOIR, G., THOMAS, G., AURIAS, A. & ROULEAU, G.A. (1993). Germline deletion in a neurofibramatosis type 2 kindred inactivates the NF2 gene and a candidate meningioma locus. *Hum. Mol. Genet.*, **2**, 1215-1220.

SATO, T., TANIGAMI, A., YAMAKAWA, K., AKIYAMA, F., KASUMI, F., SAKAMOTO, G. & NAKAMURA, Y. (1990). Allelotype of breast cancer: Cumulative allele losses promote tumor progression in primary breast cancer. *Cancer Res.*, **50**, 7184-7189.

SATO, T., SAITO, H., MORITA, R., KOI, S., LEE, J.H. & NAKAMURA, Y. (1991). Allelotype of human ovarian cancer. *Cancer Res.*, **51**, 5118-5122.

SHEFFIELD, V.C., BECK, J.S., KWITEK, A.E., SANDSTROM, D.W. & STONE, E.M. (1993). The sensitivity of single-strand conformational polymorphism analysis for the detection of single base substitutions. *Genomics*, **16**, 325-332.

TAKAHASHI, K., KUDO, J., ISHIBASHI, H., HIRATA, Y. & NIHO, Y. (1993). Frequent loss of heterozygosity on chromosome 22 in hepatocellular carcinoma. *Hepatology*, **17**, 794-799.

TROFATTER, J.A., MACCOLLIN, M.M., RUTTER, J.L., MURRELL, J.R., DUYAO, M.P., PARRY, D.M., ELDRIDGE, R., KLEY, N., MENON, A.G., PULASKI, K., HAASE, V.H., AMBROSE, C.M., MUNROE, D., BOVE, C., HAINES, J.L., MARTUZA, R.L., MACDONALD, M.E., SEIZINGER, B.R., SHORT, M.P., BUCKLER, A.J. & GUSELLA, J.F. (1993). A novel moesin-, ezrin-, radixin-like gene is a candidate for the neurofibromatosis 2 tumor suppressor. *Cell*, **72**, 791-800.

TWIST, E.C., RUTTLEDGE, M.H., ROUSSEAU, M., SANSON, M., PAPI, L., MEREL, P., DELATTRE, O., THOMAS, G. & ROULEAU, G.A. (1994). The neurofibromatosis type 2 gene is inactivated in schwannomas. *Hum. Mol. Genet.*, **3**, 147-151.

WEISSENBACH, J., GYAPAY, G., DIB, C., VIGNAL, A., MORISSETTE, J., MILLASSEAU, P., VAYSSEIX, G. & LATHROP, M. (1992). A second-generation linkage map of the human genome. *Nature*, **359**, 794-801.

YANG-FENG, T.L., HAN, H., CHEN, K., LI, S., CLAUS, E.B., CARCANGIU, M.L., CHAMBERS, S.K., CHAMBERS, J.T. & SCHWARTZ, P.E. (1993). Allelic loss in ovarian cancer. *Int. J. Cancer*, **54**, 546-551.


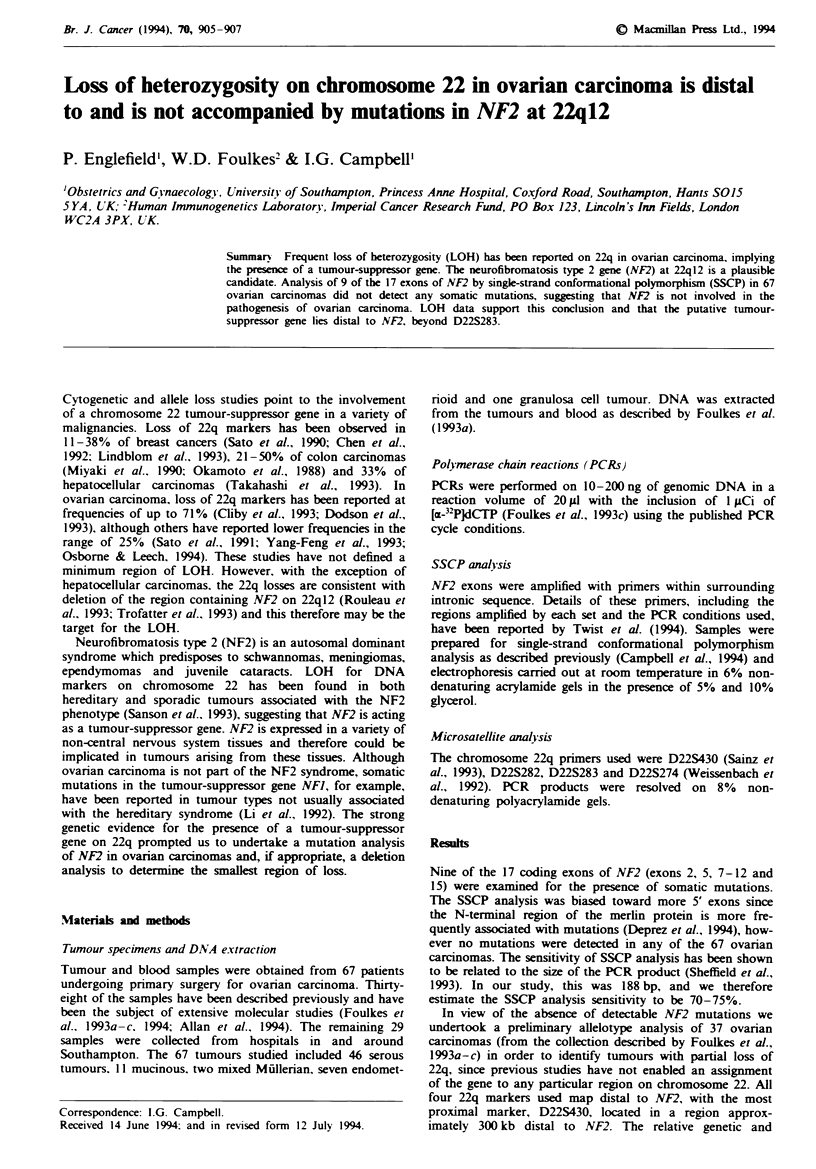

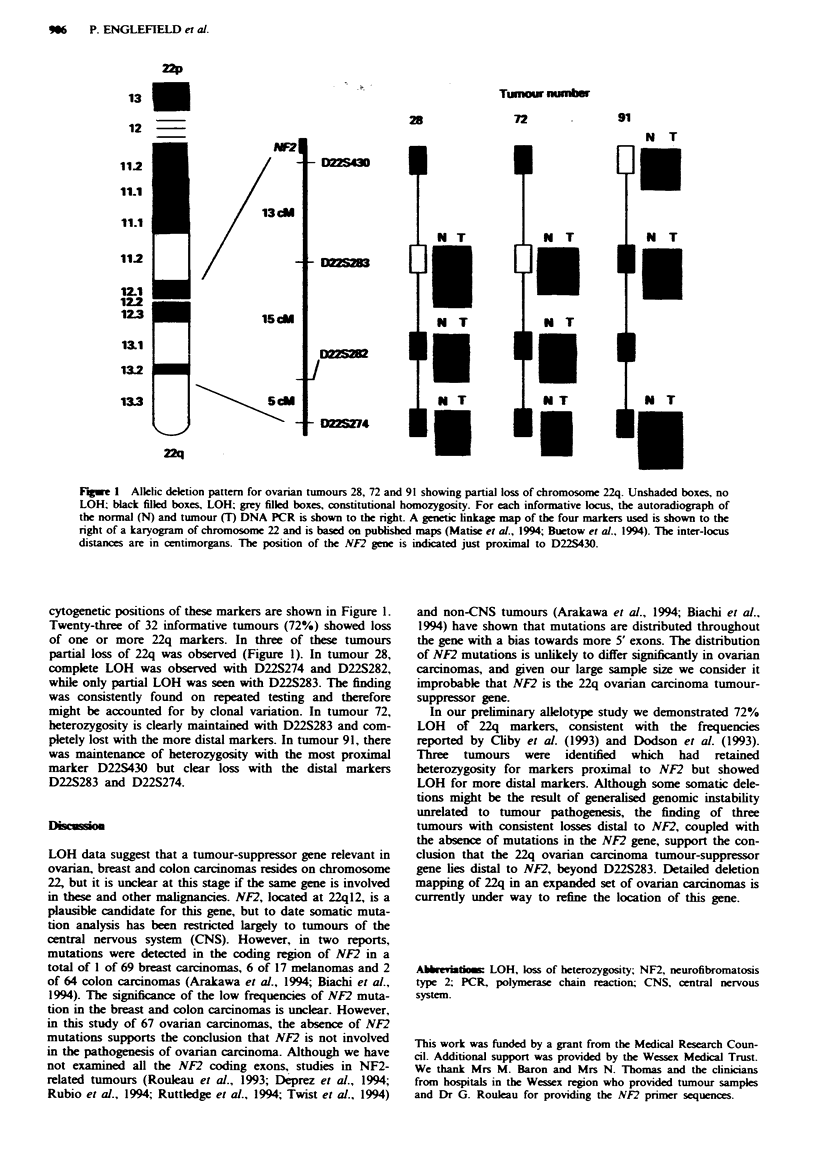

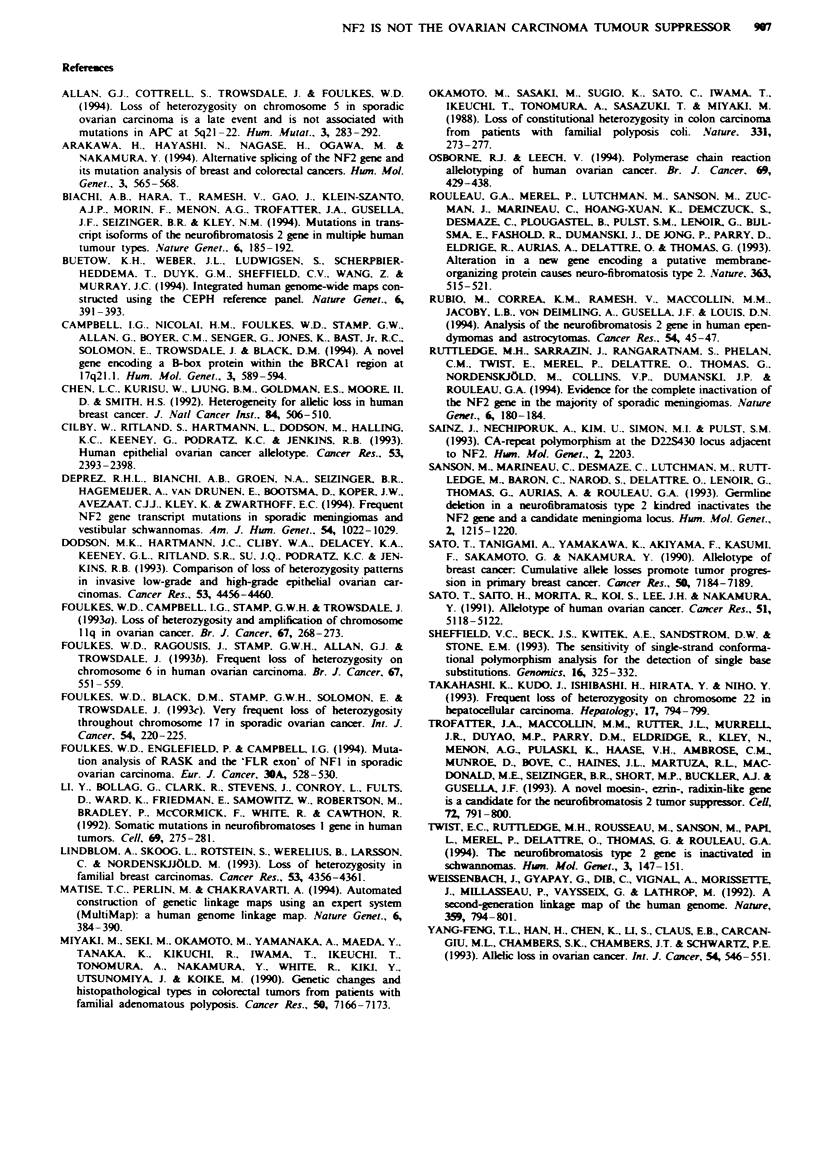


## References

[OCR_00315] Allan G. J., Cottrell S., Trowsdale J., Foulkes W. D. (1994). Loss of heterozygosity on chromosome 5 in sporadic ovarian carcinoma is a late event and is not associated with mutations in APC at 5q21-22.. Hum Mutat.

[OCR_00322] Arakawa H., Hayashi N., Nagase H., Ogawa M., Nakamura Y. (1994). Alternative splicing of the NF2 gene and its mutation analysis of breast and colorectal cancers.. Hum Mol Genet.

[OCR_00325] Bianchi A. B., Hara T., Ramesh V., Gao J., Klein-Szanto A. J., Morin F., Menon A. G., Trofatter J. A., Gusella J. F., Seizinger B. R. (1994). Mutations in transcript isoforms of the neurofibromatosis 2 gene in multiple human tumour types.. Nat Genet.

[OCR_00332] Buetow K. H., Weber J. L., Ludwigsen S., Scherpbier-Heddema T., Duyk G. M., Sheffield V. C., Wang Z., Murray J. C. (1994). Integrated human genome-wide maps constructed using the CEPH reference panel.. Nat Genet.

[OCR_00341] Campbell I. G., Nicolai H. M., Foulkes W. D., Senger G., Stamp G. W., Allan G., Boyer C., Jones K., Bast R. C., Solomon E. (1994). A novel gene encoding a B-box protein within the BRCA1 region at 17q21.1.. Hum Mol Genet.

[OCR_00348] Chen L. C., Kurisu W., Ljung B. M., Goldman E. S., Moore D., Smith H. S. (1992). Heterogeneity for allelic loss in human breast cancer.. J Natl Cancer Inst.

[OCR_00354] Cliby W., Ritland S., Hartmann L., Dodson M., Halling K. C., Keeney G., Podratz K. C., Jenkins R. B. (1993). Human epithelial ovarian cancer allelotype.. Cancer Res.

[OCR_00364] Dodson M. K., Hartmann L. C., Cliby W. A., DeLacey K. A., Keeney G. L., Ritland S. R., Su J. Q., Podratz K. C., Jenkins R. B. (1993). Comparison of loss of heterozygosity patterns in invasive low-grade and high-grade epithelial ovarian carcinomas.. Cancer Res.

[OCR_00384] Foulkes W. D., Black D. M., Stamp G. W., Solomon E., Trowsdale J. (1993). Very frequent loss of heterozygosity throughout chromosome 17 in sporadic ovarian carcinoma.. Int J Cancer.

[OCR_00371] Foulkes W. D., Campbell I. G., Stamp G. W., Trowsdale J. (1993). Loss of heterozygosity and amplification on chromosome 11q in human ovarian cancer.. Br J Cancer.

[OCR_00390] Foulkes W. D., Englefield P., Campbell I. G. (1994). Mutation analysis of RASK and the 'FLR exon' of NF1 in sporadic ovarian carcinoma.. Eur J Cancer.

[OCR_00378] Foulkes W. D., Ragoussis J., Stamp G. W., Allan G. J., Trowsdale J. (1993). Frequent loss of heterozygosity on chromosome 6 in human ovarian carcinoma.. Br J Cancer.

[OCR_00357] Lekanne Deprez R. H., Bianchi A. B., Groen N. A., Seizinger B. R., Hagemeijer A., van Drunen E., Bootsma D., Koper J. W., Avezaat C. J., Kley N. (1994). Frequent NF2 gene transcript mutations in sporadic meningiomas and vestibular schwannomas.. Am J Hum Genet.

[OCR_00393] Li Y., Bollag G., Clark R., Stevens J., Conroy L., Fults D., Ward K., Friedman E., Samowitz W., Robertson M. (1992). Somatic mutations in the neurofibromatosis 1 gene in human tumors.. Cell.

[OCR_00400] Lindblom A., Skoog L., Rotstein S., Werelius B., Larsson C., Nordenskjöld M. (1993). Loss of heterozygosity in familial breast carcinomas.. Cancer Res.

[OCR_00405] Matise T. C., Perlin M., Chakravarti A. (1994). Automated construction of genetic linkage maps using an expert system (MultiMap): a human genome linkage map.. Nat Genet.

[OCR_00414] Miyaki M., Seki M., Okamoto M., Yamanaka A., Maeda Y., Tanaka K., Kikuchi R., Iwama T., Ikeuchi T., Tonomura A. (1990). Genetic changes and histopathological types in colorectal tumors from patients with familial adenomatous polyposis.. Cancer Res.

[OCR_00422] Okamoto M., Sasaki M., Sugio K., Sato C., Iwama T., Ikeuchi T., Tonomura A., Sasazuki T., Miyaki M. (1988). Loss of constitutional heterozygosity in colon carcinoma from patients with familial polyposis coli.. Nature.

[OCR_00428] Osborne R. J., Leech V. (1994). Polymerase chain reaction allelotyping of human ovarian cancer.. Br J Cancer.

[OCR_00441] Rubio M. P., Correa K. M., Ramesh V., MacCollin M. M., Jacoby L. B., von Deimling A., Gusella J. F., Louis D. N. (1994). Analysis of the neurofibromatosis 2 gene in human ependymomas and astrocytomas.. Cancer Res.

[OCR_00451] Ruttledge M. H., Sarrazin J., Rangaratnam S., Phelan C. M., Twist E., Merel P., Delattre O., Thomas G., Nordenskjöld M., Collins V. P. (1994). Evidence for the complete inactivation of the NF2 gene in the majority of sporadic meningiomas.. Nat Genet.

[OCR_00457] Sainz J., Nechiporuk A., Kim U. J., Simon M. I., Pulst S. M. (1993). CA-repeat polymorphism at the D22S430 locus adjacent to NF2.. Hum Mol Genet.

[OCR_00460] Sanson M., Marineau C., Desmaze C., Lutchman M., Ruttledge M., Baron C., Narod S., Delattre O., Lenoir G., Thomas G. (1993). Germline deletion in a neurofibromatosis type 2 kindred inactivates the NF2 gene and a candidate meningioma locus.. Hum Mol Genet.

[OCR_00474] Sato T., Saito H., Morita R., Koi S., Lee J. H., Nakamura Y. (1991). Allelotype of human ovarian cancer.. Cancer Res.

[OCR_00471] Sato T., Tanigami A., Yamakawa K., Akiyama F., Kasumi F., Sakamoto G., Nakamura Y. (1990). Allelotype of breast cancer: cumulative allele losses promote tumor progression in primary breast cancer.. Cancer Res.

[OCR_00481] Sheffield V. C., Beck J. S., Kwitek A. E., Sandstrom D. W., Stone E. M. (1993). The sensitivity of single-strand conformation polymorphism analysis for the detection of single base substitutions.. Genomics.

[OCR_00485] Takahashi K., Kudo J., Ishibashi H., Hirata Y., Niho Y. (1993). Frequent loss of heterozygosity on chromosome 22 in hepatocellular carcinoma.. Hepatology.

[OCR_00496] Trofatter J. A., MacCollin M. M., Rutter J. L., Murrell J. R., Duyao M. P., Parry D. M., Eldridge R., Kley N., Menon A. G., Pulaski K. (1993). A novel moesin-, ezrin-, radixin-like gene is a candidate for the neurofibromatosis 2 tumor suppressor.. Cell.

[OCR_00500] Twist E. C., Ruttledge M. H., Rousseau M., Sanson M., Papi L., Merel P., Delattre O., Thomas G., Rouleau G. A. (1994). The neurofibromatosis type 2 gene is inactivated in schwannomas.. Hum Mol Genet.

[OCR_00506] Weissenbach J., Gyapay G., Dib C., Vignal A., Morissette J., Millasseau P., Vaysseix G., Lathrop M. (1992). A second-generation linkage map of the human genome.. Nature.

[OCR_00514] Yang-Feng T. L., Han H., Chen K. C., Li S. B., Claus E. B., Carcangiu M. L., Chambers S. K., Chambers J. T., Schwartz P. E. (1993). Allelic loss in ovarian cancer.. Int J Cancer.

